# High-energy blunt pelvic ring injury incidence and polytrauma caseload in a single level I trauma center during COVID-19 related pseudo-lockdown measures: a retrospective cohort study based on a prospective registry

**DOI:** 10.1186/s40001-023-01313-1

**Published:** 2023-09-08

**Authors:** Vanessa Morello, Matthieu Zingg, Elisabeth Andereggen, Alexandre Ansorge, Silvia Valisena, Axel Gamulin

**Affiliations:** 1 Division of Orthopaedic and Trauma Surgery, University Hospitals of Geneva, 4 Rue Gabrielle-Perret-Gentil, CH-1205 Geneva, Switzerland; 2 Division of Emergency Medicine, University Hospitals of Geneva, 4 Rue Gabrielle-Perret-Gentil, CH-1205 Geneva, Switzerland

**Keywords:** Pelvic ring injury, Polytrauma, High-energy trauma, Lockdown, COVID-19

## Abstract

**Background:**

Pelvic ring injuries are potentially lethal lesions associated with polytrauma patients and need an efficient trauma team for their management. The purpose of this study was to evaluate the incidence of high-energy blunt pelvic ring injuries and the absolute number of polytrauma patients in a single level I trauma center during the 2020 pseudo-lockdown period related to the Coronavirus pandemic, and to compare it with corresponding periods in 2014–2019 in order to better understand the need of organized and dedicated personnel and infrastructures.

**Methods:**

This retrospective cohort study was based on data prospectively recorded into the institutional *Severely Injured Patients’* Registry. Data were obtained for each year period (January 1st to December 31st) and corresponding pseudo-lockdown period (March 16th to June 19th). High-energy blunt pelvic ring injuries inclusion criteria were: (1) Registry entry between January 1st, 2014 and December 31st, 2020; (2) age ≥ 16 years old; and (3) pelvic ring injury presence. Corresponding exclusion criteria were: (1) death before admission; (2) transfer from another institution > 24 h after trauma; (3) penetrating, blast, burn and electrical injuries, drownings; (4) patients living outside the defined institution’s catchment area; and (5) any document attesting the patient’s will to not participate in any study. Polytrauma patients inclusion criteria were: (1) Registry entry between January 1st, 2014 and December 31st, 2020; (2) age ≥ 16 years old; and (3) Injury Severity Score ≥ 16. Corresponding exclusion criteria were: (1) death before admission; (2) transfer from another institution > 24 h after trauma; and (3) any document attesting the patient’s will to not participate in any study. Categorical variables were reported using proportions and continuous variables using medians and interquartile ranges. Because data were exhaustive for the authors’ level I trauma center, no inferential statistics were computed.

**Results:**

The incidence of high-energy blunt pelvic ring injuries and the absolute number of polytrauma patients remained within range of previous years despite pseudo-lockdown measures.

**Conclusions:**

These observations bring better knowledge about pseudo-lockdown’s impact on trauma and may help for future health strategy planning by pointing out the importance of maintaining the activity of level I trauma centers in terms of personnel and infrastructures.

**Supplementary Information:**

The online version contains supplementary material available at 10.1186/s40001-023-01313-1.

## Background

The 2020 Coronavirus disease (COVID-19) pandemic impacted the healthcare system globally and forced to introduce restrictions such as lockdown and pseudo-lockdown measures to limit inter-personal contacts and viral transmission [1–3]. These measures also prevented engaging in high-risk activities in order to decrease trauma related hospital admissions and relieve the overburden healthcare system allowing for reallocation of personnel and infrastructures [2]. Although these actions seemed logical [1, 2, 4], preliminary reports have shown confusing results regarding their efficiency [5–13]. A better understanding about the impact of these measures on trauma related hospital admissions could help for future health strategy planning.

The purpose of this study was to evaluate the incidence of high-energy blunt pelvic ring injuries (PRI) and the absolute number of polytrauma patients (PP) in a single level I trauma center during the 2020 pseudo-lockdown period related to COVID-19 pandemic and the whole 2020 year period, and to compare them with corresponding periods in 2014–2019. The hypothesis was that these restrictions reduced high-energy blunt PRI incidence and PP caseload. To the best of the authors’ knowledge, this is the first publication assessing high-energy blunt PRI incidence in a defined population during the pseudo-lockdown period.

## Methods

### Study design

This retrospective cohort study was based on data prospectively recorded into the institutional *Severely Injured Patients’* Registry (SIPR). The SIPR contains around 300 items for each patient admitted with a suspected or confirmed high-energy trauma or polytrauma. Among these items, demographic data, trauma details and mechanism, treatments, outcomes, Abbreviated Injury Scale (AIS) codes [14] and Injury Severity Scores (ISS) [15, 16] are recorded. A trained and accredited study nurse was responsible for AIS and ISS coding [14–16].

### Study setting and population

This study was conducted in a 1900-bed urban academic center serving around 500,000 inhabitants (University Hospitals of Geneva, Switzerland) and corresponding to a level I trauma center according to national and international medical authorities’ definitions [17, 18] with about 100–150 patients with an ISS ≥ 16 and 70–100 patients with an ISS ≥ 20 managed each year.

### Pseudo-lockdown measures description

Federal government issued a nation-wide pseudo-lockdown following the COVID-19 pandemic from March 16th to June 19th, 2020 [19, 20]. Measures included social distancing, quarantine, restricted travels, on-line education and office work, sports and leisure facilities’ closure, building sites’ standstill, and limited out-of-the house displacement [19]. Medical resources were mainly reallocated to COVID-19 patients. Minor trauma patients were mostly redirected to other healthcare facilities, but severely injured patients continued to be admitted at the authors’ institution.

### Participants’ characteristics

The main outcomes were the incidence of high-energy blunt PRI and the absolute number of PP. Inclusion criteria for the incidence of high-energy blunt PRI were: (1) SIPR entry between January 1st, 2014 and December 31st, 2020; (2) ≥ 16 years old; and (3) PRI presence as defined by specific AIS codes (856,100.2 and 856,101.3 corresponding to PRI not further specified; 856,151.2 and 856,152.3 corresponding to PRI with intact posterior arch; 856,161.3, 856,162.4, 856,163.4 and 856,164.5 corresponding to rotationally unstable PRI; 856,171.4, 858,172.4, 856,173.5 and 856,174.5 corresponding to vertically and rotationally unstable PRI) [14]. Corresponding exclusion criteria were: (1) death before Emergency Room (ER) admission; (2) transfer from another institution > 24 h after trauma; (3) penetrating, blast, burn and electrical injuries, drownings; (4) patients living outside the defined institution’s catchment area; (5) any document attesting the patient’s will not to participate in any study. Inclusion criteria for the absolute number of PP were: (1) SIPR entry between January 1st, 2014 and December 31st, 2020; (2) ≥ 16 year old; and (3) ISS ≥ 16 [21]. The ISS cutoff of 16 was chosen as the most common definition of PP found in the literature [21]. Corresponding exclusion criteria were: (1) death before ER admission; (2) transfer from another institution > 24 h after trauma; and (3) any document attesting the patient’s will not to participate in any study. Death before ER admission was an exclusion criterion because of the usual lack of diagnostic assessment in these cases. Transfer from another institution > 24 h after trauma was also an exclusion criterion since initial medical data may be difficult to collect. Penetrating, blast, burn and electrical injuries, as well as drownings, were exclusion criteria because these injury patterns are rare in the authors’ institution and would have added heterogeneity to the study collective. Finally, patients living outside the defined institution’s catchment area were excluded to obtain incidence values for high-energy blunt PRI which were interpreted as an indirect measure of population’s compliance to pseudo-lockdown restrictions. Alternatively, the absolute number of PP was chosen as a general indicator of the level I trauma center activity, without patients’ origin distinction—within or outside the defined catchment area.

The SIPR was used to extract age, gender, residential address (within defined catchment area or not), accident’s date, trauma mechanism, AIS codes, ISS, survival, death’s date if applicable, complications (defined as any serious secondary condition appearing during hospital stay), intensive care unit (ICU) length of stay, total acute hospital length of stay, hemodynamic instability at admission (defined as prehospital systolic blood pressure < 90 mmHg or prehospital heart rate > 100 bpm or need for packed red blood cell (PRBC) in the ER), number of PRBC units received during the first 24 h. Radiological images were reviewed by two authors and PRI classified according to the AO/OTA classification [22]. Only type B and C PRI were considered for this study as they may be associated with hemodynamic instability and usually need surgical fixation [23]. Population description of the defined catchment area of the institution was obtained from the government's *Statistics Office* [24]. Polytrauma patients were further stratified using ISS cutoffs of 20 (definition of severe trauma in Switzerland) [18] and 24 (definition of critical trauma in some publications) [25].

### Statistical analysis

The incidence of high-energy blunt PRI and the absolute number of PP were individualized for each year period (January 1st to December 31st) and the corresponding pseudo-lockdown period (March 16th to June 19th) from 2014 to 2020. Categorical variables were reported using proportions and continuous variables using medians and interquartile ranges. Because data were exhaustive for the authors’ level I trauma center, no inferential statistics were computed.

## Results

**Fig. 1:**
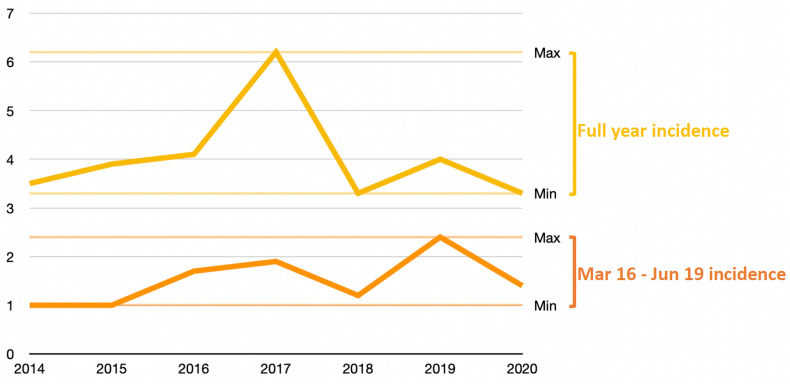
Incidence of high-energy blunt pelvic ring injuries (AO/OTA type B & C) over time. Full year incidence (yellow line and markings) and pseudo-lockdown period (March 16th to June 19th) incidence (orange line and markings) are shown for each year, with respective horizontal color lines indicating minimal and maximal incidences over the period 2014–2020

The incidence of high-energy blunt PRI during both 2020 pseudo-lockdown period and 2020 year period remained within range of previous years (Table 1 and Fig. 1). High-energy blunt PRI patients’ characteristics are shown in Table 2. Median ISS and hemodynamic instability rate remained within range of previous years for both 2020 pseudo-lockdown and year periods. Death rate was 0% for both 2020 pseudo-lockdown and year periods. The ICU rate and hospital length of stay remained within range of previous years during the 2020 pseudo-lockdown period and were lower during the 2020 year period.

**Table 1:** Absolute number and incidence of high-energy blunt pelvic ring injuries in the defined population (2014–2020)

Period	Population ≥ 16 y.o.^a^	PRI type A, B and C^b^				PRI type B and C^b^			
		Full year		Mar 16-Jun 19		Full year		Mar 16-Jun 19	
		N	Incidence	N	Incidence	N	Incidence	N	Incidence
2014	403′045	18	4.5	5	1.2	14	3.5	4	1.0
2015	409′722	21	5.1	6	1.5	16	3.9	4	1.0
2016	412′429	20	4.8	7	1.7	17	4.1	7	1.7
2017	416′254	32	7.7	9	2.2	26	6.2	8	1.9
2018	419′288	18	4.3	6	1.4	14	3.3	5	1.2
2019	423′172	19	4.5	10	2.4	17	4.0	10	2.4
**2020**	**424′901**	**16**	**3.8**	**6**	**1.4**	**14**	**3.3**	**6**	**1.4**

Values are expressed as absolute numbers except for incidence which is expressed per 100,000 persons per year

Full year corresponds to the full calendar year (January 1st to December 31st)

The period Mar 16–Jun 19 corresponds to the pseudo-lockdown period evaluated in this study: March 16th to June 19th

PRI: pelvic ring injury; Mar: March; Jun: June

The 2020 pseudo-lockdown period and year are highlighted in bold for clarity purpose

^**a**^Description of the population exclusively served by the authors' level I trauma center was obtained from the government’s *Statistics Office*

^**b**^PRI type A, B and C encompasses all types of pelvic ring injuries as described by the OTA/AO classification; PRI type B and C only encompasses type B and C pelvic ring injuries as described by the OTA/AO classification

**Table 2:** High-energy blunt type B and C pelvic ring injury patients’ characteristics (2014–2020)

Period	ISS (median, IQR1-3)		HD instability rate (%) and PRBC/24 h [median (IQR1-3)]		Death rate (%) and timing [in days: median (IQR1-3)]		ICU rate (%) and LOS [in days: median (IQR1-3)]		Hospital LOS [in days: median (IQR1-3)]	
	Full year	Mar 16–Jun 19	Full year	Mar 16–Jun 19	Full year	Mar 16–Jun 19	Full year	Mar 16–Jun 19	Full year	Mar 16–Jun 19
2014	26 (18.5–29)	31.5 (24–38)	14%; 5 (4–8)	25%; 20.5 (10.8–30.2)	14%; 11 (6–16)	25%; 1 (1–1)	50%; 12 (6.5–15.5)	25%; 22 (22–22)	16.5 (9–20.8)	10 (4.8–24)
2015	20.5 (16.8–27.2)	18.5 (15.2–22)	19%; 3 (2–4.8)	0%; *-*	0%; *-*	0%; *-*	50%; 7 (3.5–11.5)	0%; *-*	18.5 (11.8–29.5)	9 (7–11.5)
2016	29 (22–38)	24 (20–32.5)	24%; 5 (3.5–7.5)	29%; 4 (3.5–6.5)	24%; 17 (11.8–27)	29%; 26.5 (14.2–38.8)	71%; 6.5 (2–9.8)	71%; 2 (2–5)	15 (7–27)	20 (9–39)
2017	22 (17.2–40.2)	30.5 (21.5–35.8)	35%; 7 (4–10)	50%; 8.5 (6.2–10)	12%; 1 (1–26.5)	13%; 1 (1–1)	42%; 11 (8–25.5)	63%; 16 (7.8–24.5)	19 (11–52)	27 (8.5–65)
2018	29 (19.8–34)	24 (19–29)	21%; 2 (2–2)	20%; 2 (2–2)	14%; 4.5 (2.8–6.2)	0%; *-*	57%; 10.5 (3.5–17.5)	60%; 11 (11–24)	19 (13–23.8)	24 (21–75)
2019	22 (13–27)	21 (14–26.2)	35%; 12.5 (11.2–13.8)	20%; 15 (15–15)	12%; 5 (3–7)	10%; 9 (9–9)	35%; 1.5 (1–8.8)	50%; 1 (1–2)	35 (26–57)	32 (18.5–43.8)
**2020**	**22.5 (18.2–32.8)**	**27 (11.8–39.2)**	**29%; 1.5 (1–5.2)**	**33%; 1.5 (1.2–1.8)**	**0%; *-***	**0%; -**	**29%; 7 (6–13)**	**50%; 7 (5–7)**	**13 (9–19.2)**	**14 (10.2–14)**

ISS: Injury Severity Score; HD: hemodynamic; PRBC/24 h: number of packed red blood cell units received in 24 h; ICU: intensive care unit; LOS: length of stay; Mar: March; Jun: June; IQR1-3: interquartile range 1–3

Type B and C pelvic ring injury refers to the OTA/AO classification

Full year corresponds to the full calendar year (January 1st to December 31st)

The period Mar 16-Jun 19 corresponds to the pseudo-lockdown period evaluated in this study: March 16th to June 19th

The 2020 pseudo-lockdown period and year are highlighted in bold for clarity purpose

**Fig. 2:**
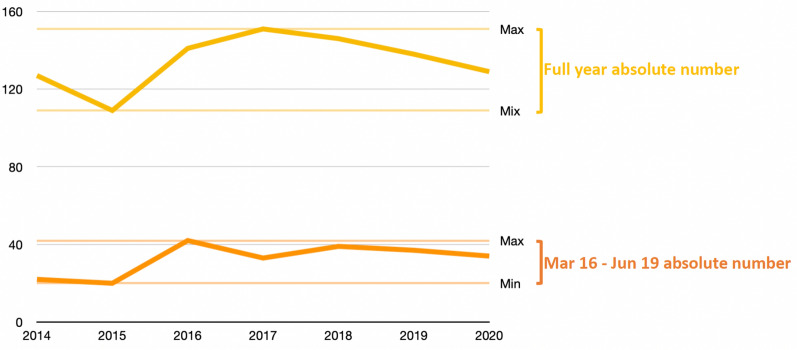
Absolute number of polytrauma patients over time. Full year absolute number (yellow line and markings) and pseudo-lockdown period (March 16 th to June 19th) absolute number (orange line and markings) are shown for each year, with respective horizontal color lines indicating minimal and maximal absolute numbers over the period 2014–2020

The absolute number of PP during both 2020 pseudo-lockdown period and 2020 year period remained within range of previous years (Table 3 and Fig. 2). The number of PP admitted with an ISS ≥ 20 or an ISS ≥ 24 was inferior to or at the lower range of previous years. Characteristics of PP are shown in Table 4. Self-inflicted trauma rate was the lowest during both 2020 pseudo-lockdown and year periods. Median ISS was lower during the 2020 pseudo-lockdown period and within range of previous years during the 2020 year period. Death rate, ICU rate, and hospital length of stay remained within range of previous years during the 2020 pseudo-lockdown period and were lower during the 2020 year period.

**Table 3:** Absolute number of polytrauma patients (2014–2020)

Period	ISS ≥ 16		ISS ≥ 20		ISS ≥ 24	
	Full year	Mar 16–Jun 19	Full year	Mar 16–Jun 19	Full year	Mar 16–Jun 19
2014	127	22	91	18	76	16
2015	109	20	77	15	60	13
2016	141	42	101	32	77	23
2017	151	33	84	24	69	19
2018	146	39	81	21	60	19
2019	138	37	90	22	66	15
**2020**	**129**	**34**	**73**	**15**	**57**	**11**

Values are expressed as absolute numbers

Patients are defined as any patient ≥ 16 year old

Polytrauma patients were defined has having an ISS ≥ 16 (common definition) and results are also presented for ISS ≥ 20 and ISS ≥ 24 as an attempt to differentiate most severe cases

Full year corresponds to the full calendar year (January 1st to December 31st)

The period Mar 16-Jun 19 corresponds to the pseudo-lockdown period evaluated in this study: March 16th to June 19th

ISS: Injury Severity Score; Mar: March; Jun: June

The 2020 pseudo-lockdown period and year are highlighted in bold for clarity purpose

**Table 4:** Polytrauma patients’ characteristics (2014–2020)

Period	Suicide as a cause of polytrauma (%)		ISS (median, IQR1-3)		Death rate (%) and timing [in days: median (IQR1-3)]		ICU rate (%) and LOS [in days: median (IQR1-3)]		Hospital LOS (in days: median (IQR1-3))	
	Full year (%)	Mar 16–Jun 19 (%)	Full year	Mar 16–Jun 19	Full year	Mar 16–Jun 19	Full year	Mar 16–Jun 19	Full year	Mar 16–Jun 19
2014	9	5	25 (18.5–29)	25.5 (22.8–29)	18%; 1 (1–5)	15%; 1.5 (1–2.8)	76%; 6 (2–13)	56%; 6.5 (2–15.8)	15 (7–25.5)	11.5 (1.2–20.2)
2015	10	15	25 (18–29)	25 (20.2–29)	12%; 1 (1–3)	15%; 1 (1–2)	75%; 4 (2–9)	57%; 2.5 (2–5.5)	13 (6–23)	9 (3–14.8)
2016	6	2	25 (18–29)	24 (20–25)	18%; 2 (2–5)	15%; 2 (1–4.2)	59%; 3 (2–7.5)	56%; 2 (2–5)	11 (4–21)	10 (2–19.8)
2017	8	12	22 (17–27)	25 (19–34)	15%; 2 (1–10.5)	15%; 1 (1–3)	56%; 5 (2–9)	57%; 6 (2–7.5)	12 (4–21)	12 (3–20)
2018	6	8	21 (17–28.5)	22 (17–28)	14%; 2 (1–6)	15%; 2 (1–3)	44%; 5 (2.8–9.2)	56%; 6 (4–8)	12 (5.2–20)	14 (6–23)
2019	7	5	22 (18–26)	22 (17–25)	16%; 3.5 (2–5.8)	15%; 4 (2.8–6.2)	45%; 4.5 (3–8)	56%; 4 (3–7)	12 (5.2–20)	9 (5–18)
**2020**	**4**	**0**	**22 (17–26)**	**19 (17–26)**	**11%; 4.5 (2.2–25)**	**15%; 4 (1.8–12.2)**	**31%; 7 (3–11)**	**55%; 5 (3–7)**	**10 (4–17)**	**9 (4–14)**

Full year corresponds to the full calendar year (January 1st to December 31st)

The period Mar 16-Jun 19 corresponds to the pseudo-lockdown period evaluated in this study: March 16th to June 19th

ISS: Injury Severity Score; ICU: intensive care unit; LOS: length of stay; Mar: March; Jun: June; IQR1-3: interquartile range 1–3

The 2020 pseudo-lockdown period and year are highlighted in bold for clarity purpose

## Discussion

This study showed no change in the incidence of high-energy blunt PRI (AO/OTA type B or C) nor in the absolute number of PP (ISS ≥ 16) in a single level I trauma center during 2020 pseudo-lockdown period and 2020 year period in comparison to previous years (2014–2019). Severity of PRI was stable when analyzing related ISS and hemodynamic instability rates. The type of PP was generally less severe with lower ISS during 2020 pseudo-lockdown period and 2020 year period in comparison to previous years.

These findings suggest that pseudo-lockdown restrictions may not have reached the desired effect in reducing the population exposure to trauma [19]. Stable ICU rate, hospital length of stay, and good survival rate during the pseudo-lockdown period suggest that sufficient resources were allocated to high-energy PRI patients. Lower ICU rate and hospital length of stay during the 2020 year period could be due to prompter management as a consequence of the overburden healthcare system, without negative impact on survival rate.

Despite other authors having highlighted a decrease in PP admissions during lockdown period [9–11], the present study did not show comparable results. Some other investigations came to the same conclusion [7, 12, 13] and even reported an increase in PP cases [5, 6]. A possible explanation might be that the authors’ institution was the only level I trauma center serving the region and no reorganization of polytrauma management was made during pseudo-lockdown; thus, some less severe PP were not re-directed to level II trauma centers. Interestingly polytrauma cases were less severe with lower ISS as reported by Chiba et al. [10]. This might be related to population’s behavioral adaptations to restrictions, with potentially less road traffic accidents and changes in trauma etiology linked to illicit substance use, self-inflicted trauma, and interpersonal violence [8, 10]. Data available for the present study did not allow further analysis of trauma mechanism and cause, except for self-inflicted injury rates which were lower for both 2020 pseudo-lockdown period and 2020 year period. This was in contradiction with other studies having highlighted higher rates of self-inflicted injuries, possibly linked with emotional stress due to isolation [8, 10]. Stable death rate, ICU rate, and hospital length of stay during the pseudo-lockdown period might indicate that despite the burden of COVID-19 patients management, sufficient resources were allocated for PP allowing stable survival rates. Decreased death rate, ICU rate, and hospital length of stay during the 2020 year period might be related to their lower ISS.

Analysis of both main outcomes showed no decrease in exposure to trauma nor in the level I trauma center activity during the pseudo-lockdown period. This is the first publication highlighting the stability of the incidence and severity of high-energy blunt PRI during the 2020 pseudo-lockdown period and 2020 year period in comparison with previous years in a single level I trauma center.

This study had several limitations. Its retrospective design made it prone to several biases, despite data being prospectively collected. Due to its monocentric setting, conclusions might apply only to the authors’ institution. Furthermore, polytrauma definition as ISS ≥ 16 might not be universally accepted [26], but it was constant throughout the whole study period.

## Conclusions

During the 2020 pseudo-lockdown period, no decrease was found in the incidence of high-energy blunt PRI nor in the absolute number of PP in the authors’ level I trauma center demonstrating no change in its activity. These observations bring better knowledge about pseudo-lockdown’s impact on trauma and may help for future health strategy planning by pointing out the importance of maintaining the activity of level I (and possibly level II and III) trauma centers in terms of personnel and infrastructures.

### Supplementary Information

**Additional file 1. **Polytrauma patients database.

**Additional file 2. **Pelvic ring injury patients database.

## Data Availability

All data generated or analyzed during this study are included in this published article and its Additional files 1, 2.

## Abbreviations

AIS: Abbreviated Injury Scale
AO/OTA: Arbeitsgemeinschaft für Osteosynthesefragen/Orthopaedic Trauma Association
COVID-19: Coronavirus disease 2019
ER: Emergency Room
ICU: Intensive Care Unit
ISS: Injury Severity Score
PRBC: Packed Red Blood Cell
PRI: Pelvic Ring Injuries
PP: Polytrauma Patients
SIPR: Severely Injured Patients’ Registry
